# Effects of Infrared Optical Trapping on *Saccharomyces cerevisiae* in a Microfluidic System

**DOI:** 10.3390/s17112640

**Published:** 2017-11-16

**Authors:** Zdeněk Pilát, Alexandr Jonáš, Jan Ježek, Pavel Zemánek

**Affiliations:** 1 Institute of Scientific Instruments of the CAS, v.v.i., Czech Academy of Sciences, Královopolská 147, 612 64 Brno, Czech Republic; jezek@isibrno.cz (J.J.); pavlik@isibrno.cz (P.Z.); 2 Department of Physics, Istanbul Technical University, Maslak, 34469 Istanbul, Turkey; ajonas1972@gmail.com

**Keywords:** optical trapping, microfluidics, phototoxicity, laser, *Saccharomyces cerevisiae*

## Abstract

Baker’s yeast (*Saccharomyces cerevisiae*) represents a very popular single-celled eukaryotic model organism which has been studied extensively by various methods and whose genome has been completely sequenced. It was also among the first living organisms that were manipulated by optical tweezers and it is currently a frequent subject of optical micromanipulation experiments. We built a microfluidic system for optical trapping experiments with individual cells and used it for the assessment of cell tolerance to phototoxic stress. Using optical tweezers with the wavelength of 1064 nm, we trapped individual *Saccharomyces cerevisiae* cells for 15 min and, subsequently, observed their stress response in specially designed microfluidic chambers over time periods of several hours by time-lapse video-microscopy. We determined the time between successive bud formations after the exposure to the trapping light, took account of damaged cells, and calculated the population doubling period and cell areas for increasing trapping power at a constant trapping time. Our approach represents an attractive, versatile microfluidic platform for quantitative optical trapping experiments with living cells. We demonstrate its application potential by assessing the limits for safe, non-invasive optical trapping of *Saccharomyces cerevisiae* with infrared laser light.

## 1. Introduction

Baker’s yeast (*Saccharomyces cerevisiae*) and its ability of fermentation gave rise to the first microbial biotechnology ever known to human kind probably around 10,000 years ago [1]. From those prehistoric times, the importance of this unicellular fungus has grown ever larger. Historically, there is no microorganism more biotechnologically important for human society. Baker’s yeast genome was the first eukaryotic genome that was completely sequenced [2]. Currently, baker’s yeast is the largest source of our knowledge of eukaryotic cellular machinery down to the level of individual protein interactions [3]. It was also among the first living microorganisms manipulated by a focused laser beam soon after the demonstration of single-beam optical trapping by Arthur Ashkin [4]. 

Later on, more sophisticated studies using optical experimental techniques followed, e.g., identification of dead and living optically trapped yeast cells by Raman spectroscopy [5], spatially-resolved confocal Raman spectroscopy [6], and combined optical trapping and laser microsurgery of living yeast cells [7]. Many publications involving optical trapping of *S. cerevisiae* concentrated on assessment of various stress conditions, such as hyperosmotic stress [8,9], oxidative stress [10], or glucose availability [11]. Other studies focused on the adhesion properties of yeast cells and measurement of the forces necessary for the cell removal from the substrate [12,13]. Optical trapping was also used for yeast cells sorting in a microfluidic system [14] or isolation of vacuole-lacking mutants of yeast from the rest of the population [15]. Depolymerization of actin cytoskeleton was observed in real time in a single optically trapped yeast cell [16]. Dual-trap Raman tweezers were used for Raman-probing of the yeast budding process in an individual trapped cell [17]. Growth patterns of individual yeast cells were observed in a line optical trap [18]. However, despite the abundance of optical trapping experiments with *S. cerevisiae*, including routine isolation of selected cells [19], phototoxicity of optical trapping for yeast cells received rather limited attention [13,20,21]. Additional studies of detrimental effects of optical trapping on living cells have been carried out with both prokaryotic [22] and multicellular eukaryotic [23] model systems.

Here, we report our experiments in which we test the tolerance of baker’s yeast cells to optical trapping by a single beam trap formed by a tightly focused laser beam at 1064 nm wavelength. Ever since the introduction of optical trapping into cell and molecular biology and biophysics almost thirty years ago, lasers operating in the near-infrared spectral region (wavelengths around 1 μm) have been the most popular experimentalist’s choice, as the light in this part of the spectrum is relatively weakly absorbed by water and intracellular chromophores. In addition, near-infrared light is well transmitted through commonly used microscope objectives and laser sources with a sufficiently high output power are largely available. In order to study the effects of near-infrared optical trapping on the yeast cells, we apply an experimental procedure similar to that used previously to analyze the impact of optical trapping on photosynthetic cells of unicellular algae *Trachydiscus minutus* [24]. We take advantage of microfluidic chips equipped with arrays of micro-chambers which allow us to study the stress response of yeast cells induced by laser trapping in terms of generation time and mortality of the cells at the single-cell level. Multiple micro-chambers with identical dimensions fabricated in the same chip enable us to keep the control cells spatially separated from irradiated individuals. This facilitates quantitative analysis of the cell division dynamics, as the populations of daughter cells originating from individual mother cells do not mix with each other. Moreover, separate micro-chambers prevent the control cells from accidentally entering the optical trap and limit the diffusive transport of chemical signals between the neighboring cells that could potentially influence the experimental results. At the same time, all the cells in the chip can be maintained at identical environmental conditions, thus providing a robust reference for the mortality, cell area and generation time in the absence of light-induced stress. Optical trapping of yeast cells by 1064 nm light for 15 min at 19 mW of power is found to cause no delay in reproduction or increased mortality, although it reduces the mean cell size. Under otherwise identical experimental conditions, trapping with 38 mW of laser power causes significant delay in reproduction and marginal mortality, while 76 mW and 95 mW of trapping power result in 50% and 90% mortality, respectively. The presented research utilizes a microfluidic platform designed for quantitative single-cell experiments for testing the safe boundaries of non-invasive optical micromanipulation of individual cells of *S. cerevisiae* with infrared laser light.

## 2. Materials and Methods

**Figure 1:**
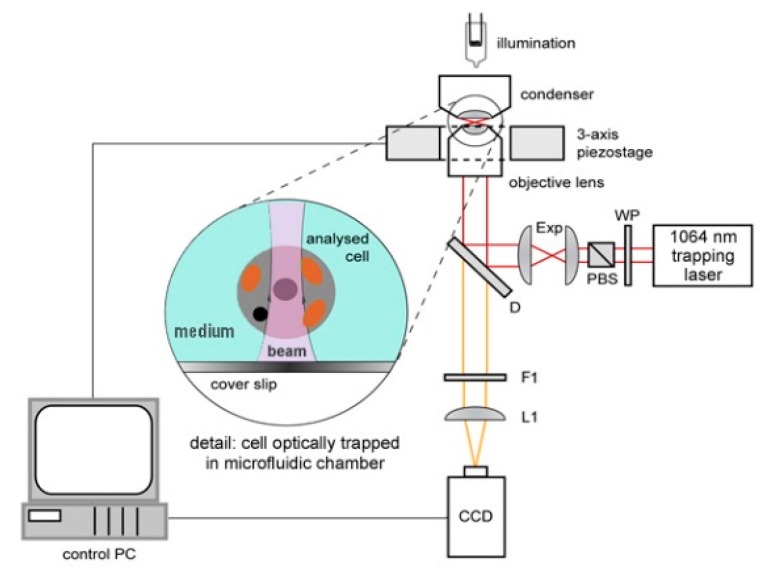
Experimental setup for optical trapping and video microscopy of *Saccharomyces cerevisiae* cells. WP: half-wave-plate; PBS: polarizing beam splitter; Exp: expander; D: dichroic mirror; CCD: CCD camera; F1: edge filter; L1: focusing lens. For detailed parameters of the system components, see the main text.

The experimental setup for observation of optically trapped yeast cells using time-lapse video microscopy is depicted in Figure 1. Infrared trapping laser beam (1064 nm, diode pumped Nd:YAG; DPY 321 II, Adlas, Lubeck, Germany) was introduced into the system through a half-wave-plate (WP, AHWP10M-980, Thorlabs, Newton, NJ, USA) and a polarizing beam splitter (PBS, PBS201, Thorlabs, Newton, NJ, USA). These optical elements provided fine-tuning of the laser power incident on the trapped cell. Expander (Exp) constructed from two achromatic lenses (C240TM-C, f = 8 mm and AC254-25-C, f = 75 mm, Thorlabs, Newton, NJ, USA) was used to obtain a wide collimated beam which was reflected from a dichroic mirror (D, highly reflective at wavelengths above 785 nm; made in ISI CAS, Brno, Czech Republic) into a microscope objective lens with a high numerical aperture (UPLSAPO, 60×, NA 1.20, Olympus, Tokyo, Japan) which created the actual optical trap. White light for sample illumination was focused on the sample by a condenser, collected by the objective lens and, after passing through the dichroic mirror, it was focused on a standard CCD camera (piA1600, Basler, Ahrensburg, Germany) with an achromatic tube lens (L1, AC508-150-B-ML, f = 150 mm, Thorlabs, Newton, NJ, USA). Overall magnification of the imaging optical system was chosen so as to image simultaneously a single irradiated (optically trapped) cell and two non-irradiated reference cells located in adjacent micro-chambers in each experiment. In order to block the infrared trapping light in the images, an edge filter (F1, highly reflective at 1064 nm, made in ISI CAS, Brno, Czech Republic) was adopted.

The laser power in the sample plane *P_s_* was determined by measurement of the laser power before the microscope objective *P_m_* and subsequent multiplication by the transmittance of the objective *τ_obj_*: $P_{s}=P_{m}\cdot \tau_{obj}$. The transmittance of the objective *τ_obj_* was assessed by the dual-objective transmittance technique [25] in which a laser beam is sent through two oppositely facing objectives with identical parameters, aligned along the same optical axis. The input power *P_i_* of the laser beam was measured before entering the first objective and the output power *P_o_* was measured after passing through both objectives. The single-objective transmittance *τ_obj_* could then be calculated as $\tau_{obj}=\sqrt{P_{o}/P_{i}}$. The transmittance of the objective used in our experiments was measured to be *τ_obj_* = 0.38 at the trapping wavelength. The cover glass transmittance *τ_cov_* was determined to be *τ_cov_* > 0.9 and it was omitted in the calculation of the power in the sample plane, since it represented a negligible error. Due to the high numerical aperture of the used microscope objective, the trapping laser beam focus was significantly smaller than the typical diameter of *S. cerevisiae* cells. Therefore, the laser power in the sample plane *P_s_* was assumed to be equivalent to the trapping laser power incident on the cell *P_c_*: $P_{c}≅P_{s}=P_{m}\cdot \tau_{obj}$. The laser energy *E_c_* delivered to the cell over the course of the optical trapping time was calculated from the trapping time *t_ot_* in seconds and *P_c_* in watts: $E_{c}=P_{c}\cdot t_{ot}$.

Our microfluidic chips were fabricated from poly(dimethyl)siloxane (PDMS) by conventional soft lithography, using master stamps based on negative SU-8 epoxy photoresist deposited on a silicon substrate. In brief, SU-8 was spin-coated on the silicon wafer, illuminated by a UV lamp through a mask, and developed. The masks for photolithographic patterning of SU-8 were fabricated by inkjet printing on a transparent foil by a specialized company (Gatema, Brno, Czech Republic). PDMS mixture (base to curing agent ratio of 10:1) was then poured into a mold formed by the SU-8 master stamp on Si wafer at the bottom and a square frame machined form polycarbonate. After curing, the resultant PDMS device was peeled off from the mold and attached to a glass slide using standard oxygen plasma treatment.

**Figure 2:**
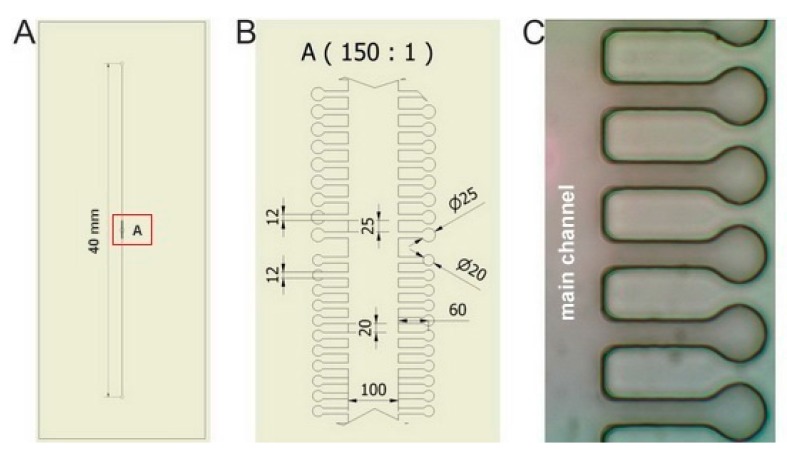
Microfluidic chip used in the optical trapping experiments. (**A**)—The design of the microfluidic chip. Total length of the main channel: 40 mm; (**B**)—a detail of the central part of the chip contained within the red rectangular region shown in part A (dimensions in µm); (**C**)—a microscope image of individual micro-chambers in the chip and adjacent main channel.

The layout of microfluidic chips used in the experiments is apparent from Figure 2. Individual sample chambers of cylindrical shape (diameter 20 μm or 25 μm) were connected to the wide main microfluidic channel (width 100 μm) by side channels of width 12 μm and length 60 μm. Height of all chambers and channels in the chip was 20 μm. Such configuration ensured that the cells could not escape easily from the chambers only due to their diffusion. On the other hand, the length of the side channels was sufficiently short to permit diffusion-mediated replenishment of nutrients in the chambers during the course of the experiment. The complete microfluidic system consisted of a syringe pump (NE1001, New Era Pump Systems, Inc., Farmingdale, NY, USA), a 1 mL glass syringe (Hamilton, Bonaduz, Switzerland), a luer-lock connector (IDEX Health & Science LLC, Oak Harbor, WA, USA), and microfluidic tubing from the same manufacturer (PEEK, internal diameter 360 μm), which connected the chip to the syringe on one end of the main channel and to a waste container on the opposite end. In all experiments, flow rate of the cultivation medium was set to 100 μL/h.

Baker’s yeast (*S. cerevisiae*) was provided by Lesaffre (Marcq-en-Baroeul, France) and cultivated in YPD (yeast extract, peptone, dextrose) medium (glucose 20 g, yeast-extract 10 g, tryptone 20 g, tap water 1 L, sterilized by autoclaving for 15 min at 120 °C). A sample of the fresh pressed yeast was collected with a 50 μL bacteriological loop, suspended in 5 mL of YPD medium and incubated with shaking at room temperature (22 °C) for 60 min before injection to the chip. Variations in the cell count of the injected culture had no influence on the experiment. 

**Figure 3:**
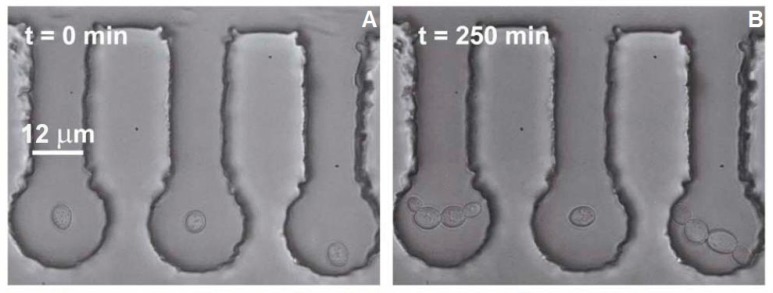
Dynamics of division of *Saccharomyces cerevisiae* cells stressed by optical tweezers in a microfluidic chip. The cell in the middle micro-chamber was optically trapped with laser power high enough to halt the division, while the two peripheral control cells continue budding. Part (**A**) shows the beginning of the experiment; Part (**B**) demonstrates repeated budding of the peripheral cells 250 min later.

The procedure for optical trapping experiments with yeast cells was as follows. First, the cell culture suspended in the YPD medium was introduced into the main microfluidic channel. Subsequently, all cells studied in a single experimental run were placed one-by-one into adjacent micro-chambers using low-power optical tweezers. In order to minimize the impact of optical trapping on the cells, we adjusted the laser power near the minimal effective trapping power (approx. 10 mW). In addition, this initial optical manipulation was carried out as quickly as possible (in less than 10 s). Figure 3A illustrates the starting configuration before each experimental run; as seen in this figure, all analyzed cells were well isolated from the bulk of the cell culture. The cell in the central chamber was then subjected to the 1064 nm focused trapping beam for *t_ot_* = 15 min, with the beam power at the sample plane *P_s_* varying between 19 mW and 95 mW. In all experiments, irradiated cells were accompanied by one or two control cells in the adjacent sample chambers that were not exposed to the high-power trapping beam. During the experiment, a slow stream of YPD medium was allowed to perfuse the main microfluidic channel to provide nutrients for unrestricted cell growth in the chambers and to wash away the surplus cells out of the main channel. Additionally, constant flow of the culturing medium assisted with dissipating unwanted heat developed in the sample due to irradiation with the focused trapping laser beam. Activity of the cells over time periods up to 9 h was recorded on a CCD camera in a time-lapse mode, collecting 1 frame every 5 s, and the video-files were analyzed off-line in order to assess the *mortality* (M), the *generation time* (GT), and the *cell area index* (CAI). 

The cells which ruptured or did not change their volume significantly and did not resume budding for more than 6 h after the optical trapping had finished were collectively termed “dead” cells. All data from the experimental runs where the control cells did not start budding were discarded. The proportion of the dead cells in the studied population was termed *mortality*. On the basis of the analysis of time-lapse image sequences, the value of M was calculated as the relative fraction of the number of the dead cells *N_d_* to the sum of both dead *N_d_* and living *N_l_* cells within the experimental group irradiated with the same trapping laser power, which can be summarized as:

$$M=\frac{N_{d}}{N_{d}+N_{l}}\cdot 100$$

The *generation time* was defined as the average time elapsed between the *time of the first bud initiation* *t_b_*_1_ and the *time of the second bud initiation t_b_*_2_ for the experimental group of *n* cells irradiated with the same trapping laser power:

$$\mathrm{GT}=\frac{1}{n}\overset{n}{\underset{i=1}{\sum }}{\left(t_{b2}-t_{b1}\right)}_{i}$$

**Figure 4:**
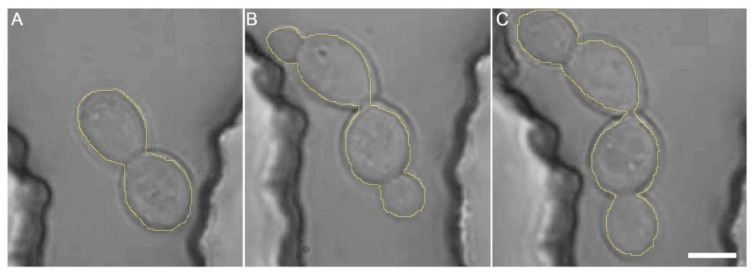
Measurement of area occupied by budding *Saccharomyces cerevisiae* cells. Time-lapse images of a budding yeast cell are shown in 30 min increments from (**A**–**C**). The area of the cells in terms of the number of pixels was measured manually on the images by marking the circumference of the cell mass (yellow line) and calculating the area of circumference region in ImageJ software. Scale bar length: 5 μm.

The cell area was obtained from the video sequences by determining the number of pixels occupied by cells in 30 min intervals ranging from 0 to 240 min after optical trapping had finished. The measurements were realized in ImageJ software (NIH; see Figure 4 for illustration). The error of the cell-area measurements was estimated by manual image analysis to be below 5%. The *cell area index* at time *t* was calculated as the ratio of the number of image pixels $N_{p}\left(t\right)$ occupied by cells at time *t* and the number of image pixels $N_{p}\left(t_{0}\right)$ occupied by cells immediately after optical trapping had finished, at time *t*_0_:

$$\mathrm{CAI}\left(t\right)=\frac{N_{p}\left(t\right)}{N_{p}\left(t_{0}\right)}$$

In total, 79 cells were examined including 53 cells optically trapped at various trapping powers and 26 reference cells.

## 3. Results and Discussion

**Figure 5:**
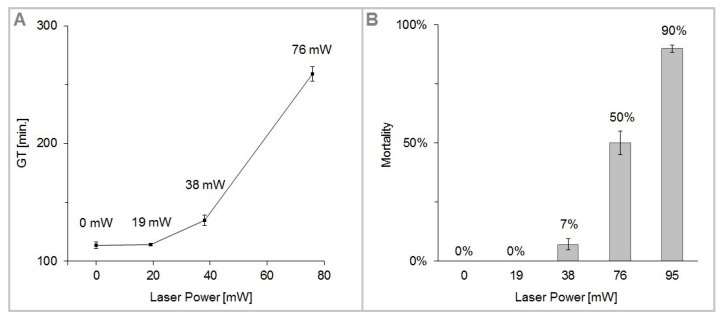
(**A**): Generation time (GT) of *S. cerevisiae* cells trapped for 15 min in optical tweezers with laser wavelength 1064 nm and trapping laser power *P_c_* in the range from 19 to 95 mW. The 0 mW point corresponds to the control unexposed cells; (**B**): mortality (M) of optically trapped *S. cerevisiae* cells under the experimental conditions identical to those in the generation time plot. On average, 13 samples were used for each data point. Error bars correspond to the standard error of the mean (SEM).

The values of GT, M and CAI obtained from our experiments are summarized in Table 1, which also includes a section presenting the data of Aabo et al. [20] for direct quantitative comparison. To further facilitate the comparison of the two experimental data sets, we have calculated from our data the quantities *µ_max_* (maximal growth rate) and DT (doubling time) according to the methodology used by Aabo et al. In brief, maximal growth rates *µ_max_* were obtained from exponential fits of the time dependence of CAI in the form $\mathrm{CAI}\left(t\right)=\exp\left(\mu_{max}t\right)$. Subsequently, doubling times DT were determined as $\mathrm{DT}=\ln2/\mu_{max}$. Figure 5 shows the plots of GT and M as a function of the trapping laser power *P_c_*. We have found the cells of *S. cerevisiae* highly tolerant to optical trapping powers up to *P_c_* = 38 mW for *t_ot_* = 15 min (laser energy *E_c_* = 34 J), which resulted in only about 7% dead cells and less than 20% increase of the cell generation time. In the interval of the trapping power *P_c_* from 76 mW to 95 mW, cell mortality M rose sharply from 50 to 90%. For the given experimental conditions (cells growing in YPD medium at the ambient temperature of 22 °C with moderate oxygen availability), the generation time was 114 min in non-trapped control cells, while at the trapping power *P_c_* = 76 mW it was 259 min, which is 228% of the control value. The irradiated cells did not divide within the duration of the experiment with the trapping power *P_c_* = 95 mW. 

**Figure 6:**
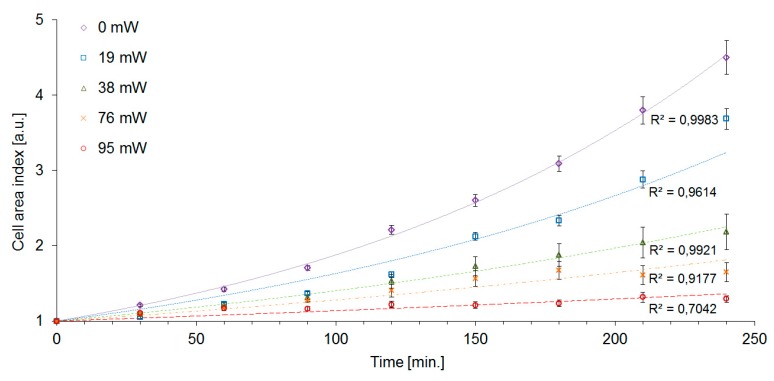
Cell area index (CAI) of *S. cerevisiae* cells trapped for 15 min in optical tweezers at the wavelength of 1064 nm using the trapping laser power *P_c_* in the range from 19 mW to 95 mW. The 0 mW curve corresponds to the control unexposed cells. The data were fitted with exponential trend lines. Error bars represent the standard error of the mean (SEM).

The cell area index CAI was significantly affected by laser trapping even with the lowest laser power *P_c_* = 19 mW. At these trapping conditions, we determined CAI(240) = 3.7, i.e., 80% of the value measured with control unexposed cells, although the generation time GT and the mortality M were virtually unaffected at the same trapping power (see Table 1). While the control cells increased their area by a factor of CAI(240) = 4.5 after 240 min, the highest intensity of the trapping laser (*P_c_* = 95 mW) caused the cells to slow the growth down to CAI(240) = 1.3. CAI measurements in time intervals of 30 min allowed us to construct the cell growth curves which are shown in Figure 6. The exponential character of the cell growth was apparent in control cells and with the trapping powers up to *P_c_* = 38 mW. In the experimental variants with the trapping power *P_c_* of 76 mW and 95 mW, the growth curves displayed more sub-exponential character. Overall, CAI was found to be the most sensitive indicator of cell stress that was employed in our study. Nevertheless, in order to obtain a more complete picture of the processes triggered in yeast cells by optical trapping, it is still useful to evaluate the other characteristics of the cell growth, in particular, DT and GT. In our experiments, the measured values of GT of irradiated cells were systematically lower than the values of DT (see Table 1). This result implies that the daughter cells produced by subsequent buddings are generally smaller than the original mother cells. In addition, recorded generation time also increased with increasing trapping power, indicating slowing-down of the cell division rate with increasing light exposure. From the plot of CAI given in Figure 6, it is obvious that larger doses of the trapping laser light slow down the growth of the overall accumulated cell mass. However, this plot alone does not discriminate between the situation in which the cells just grow more slowly but reach the same terminal size, the situation in which the generation time is the same but the daughter cells are consistently smaller, and the situation where both effects take place simultaneously. Different effects of laser-induced stress on CAI and GT could be possibly interpreted as the result of laser light having different interactions with metabolic products or signaling and metabolic pathways necessary for cell growth and those necessary for cell division, leading to a certain degree of selectivity in the process.

**Figure 7:**
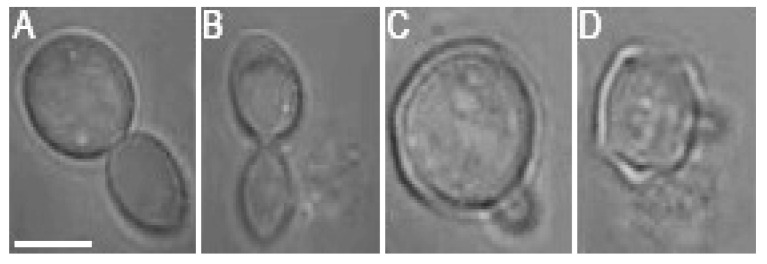
Images of two different *S. cerevisiae* cells before (**A**,**C**) and after (**B**,**D**) cell-wall rupture caused by 15 min of optical trapping at the wavelength 1064 nm with trapping laser power *P_c_* = 76 mW. The rupture always occurred during the new bud formation, more than 60 min after the end of optical trapping. Scale bar length: 5 μm.

While the negative effects of optical trapping might fade in the progeny of the trapped cell, upon crossing a certain threshold, the damage accumulated in the mother cell and subsequently transferred to the progeny (bud) will be too serious for the daughter cell to survive. It was observed in several cases that budding was initiated during the optical trapping, regardless of the laser power incident on the cell. However, the budding at *P_c_* values of 76 mW and 95 mW was usually aborted and followed by a rupture of the cell wall, regardless of whether the budding was initiated during or after the optical trapping period (see Figure 7 for illustration). Budding cell rupture during the optical trapping was typically observed at *P_c_* = 95 mW while a delayed rupture of budding cells was observed repeatedly in the samples studied at *P_c_* = 76 mW. 

We compared our experimental results with those obtained previously by Aabo et al. [20]. In their study, they irradiated the cells with a 1070 nm continuous-wave laser either continuously for several hours or in relatively short (less than 1 h) light “pulses” with duration and power varied in such a way that the total dose received by the cells remained constant. In essence, the pulsed-mode experiments of Aabo et al. are identical to our experimental protocol, except that we applied varied doses of the trapping light to the studied cells. We can safely assume the difference in the trapping light wavelength used in the two studies (6 nm) is negligible in terms of absorption in the sample. In the experiments reported by Aabo et al., 5 min exposure with 10 mW of laser power uniformly spread over the cell resulted in an increase of the generation time from 92 min (control) to 139 min (irradiated) representing 151% of the control value, and the cells continued growing exponentially. In contrast, our cells exposed to the trapping beam power *P_c_* = 19 mW for 15 min received almost 6 times more laser energy *E_c_* and still continued growing exponentially while the generation time increased only by 1%, from 114 min to 115 min. After exposing the cells to continuous 5-h irradiation by 2 mW of 1070 nm light, Aabo et al. observed increased generation time similar to that measured by us at a much higher trapping power *P_c_* = 76 mW (GT ~ 250 min). While in our case, the time of trapping was 20 times shorter, our trapping power was 40 fold higher, resulting in twice the total laser energy *E_c_* passing through the cell and still leading to the same results in terms of the cell generation time. Taken together, these experiments suggest that the exposure of cells to low laser power over long time periods is more deleterious for cells than a short exposure with a much higher light intensity. Moreover, regardless of the exposure time and irradiation mode (continuous vs. pulsed), irradiation with a uniform light wave appears to be more harmful for the yeast cells than the exposure with a tightly focused optical trap.

The reasons for apparently lower phototoxicity of focused infrared trapping light observed in our experiments, compared to the plane-wave illumination used by Aabo et al., can be speculated about. Since the focused beam spot is smaller than the typical cell size, it might not influence the cell volume as a whole, which might be important for proper functioning of certain parts of cellular machinery, such as the cytoskeleton in cortical areas of the cell, or the elements of the cell wall. In our experiments, we used a water-immersion objective lens with the numerical aperture NA = 1.2 to create the optical trap. Thus, the radius r of the diffraction-limited focal spot for the trapping wavelength λ=1064 nm can be estimated as r=0.61λ/NA≈0.54 μm. Along the beam axis, the size Δz of the focal volume is approximately $\Delta z=4n_{e}\lambda /{\mathrm{NA}}^{2}\approx 3.9\;\mu m$, taking into account the refractive index of water $n_{e}=1.33$. Since the typical diameter D of our yeast cells is around 5 μm (see Figure 3 and Figure 4), the ratio $R_{V}$ of the irradiated volume to the cell volume is approximately $R_{V}=\left(\pi r^{2}\Delta z\right)/\left(\pi D^{3}/6\right)\approx 0.055$, assuming a spherical cell and a cylindrical focal volume. Thus, only about 5% of the cell volume is exposed to the trapping light, as opposed to 100% exposure in the experiments of Aabo et al. It might be useful to repeat the experiments with cell-wall mutants of *S. cerevisiae* to establish whether they are more susceptible to the photodamage. This might also elucidate the mechanism of the cell rupture during the optical trapping and of the delayed cell rupture. Regarding the deleterious effect of the long term, low power exposure in comparison with the short term, high power trapping, the reparative mechanisms might play a role, enabling the cell to resume normal functions effectively after a short exposure to a high intensity beam. Also, long exposure time may cause direct interference with the mitotic spindle formation and therefore substantially influence the generation time. We are planning to realize optical trapping of GFP fusion cell lines containing GFP-tubulin in order to gauge the cytoskeletal impairment. GFP-caspase could be similarly useful in linking the phototoxicity to apoptosis. 

The mechanisms of the cell cycle inhibition and cell destruction from optical trapping are multiple. They involve heating of the cell [26,27], creation of reactive oxygen species, photo-inactivation of proteins, or absorption by pigments [28]. According to Liu et al. [26] and Peterman et al. [27], heating induced by IR optical tweezers in aqueous environment is about 10 °C per watt of trapping power. This is not nearly enough to kill the yeast cells with our levels of the trapping power. Even in our case, in the enclosed glass–PDMS chamber filled with aqueous culturing medium, one would not expect the temperature increase due to the laser light absorption in water, glass, or PDMS to be large enough to kill the cells or inhibit the cell growth. However, some cell components, such as pigments, may absorb enough energy to induce a heat stress response. One should keep in mind that the trapped cells were exposed to this heating only for 15 min, after which the conditions for all cells (previously trapped and controls) were identical and they all continued growing at the same temperature. Heat-Shock Proteins (HSP, molecular chaperones) are some of the cellular components known to provide tolerance to increased temperatures (such as those encountered in infrared laser trapping) [29]. In order to resolve the relative importance of heat generated during the optical trapping for the dynamics of the yeast cell growth, we plan to repeat our experiment with heat sensitive mutants of *S. cerevisiae* and adopt sample chambers with externally adjustable temperature. Additional possibilities for controlling the local temperature of the sample might include the use of a trapping laser wavelength that is absorbed differently in the aqueous culturing medium than our present wavelength of 1064 nm or the preparation of the cell culturing medium from deuterium oxide (D_2_O) that absorbs significantly less light at 1064 nm. 

The cells used in our experiments were selected to have similar size and no buds at the time when the trapping was started. Therefore, it is most probable that the optically trapped and control cells were either in lag phase or G_1_ phase of their cell cycle, which is consistent with the absence of buds followed by budding initiation after the trapping had been completed. It is quite likely that the cells in different phases of the cell cycle respond variably to the stress induced by the laser light. Some insight into these response mechanisms could be provided by experiments with optical trapping of yeast cells synchronized in different phases of the budding process. Some experiments have already been realized with combination of optical trapping and Raman spectroscopy [30]. In the experiments reported in this article, the cells were not synchronized; this fact is directly reflected in the error bars of data points presented in Figure 5 and Figure 6. However, despite the lack of cell synchronization, the observed trends are still quite clear and unambiguous. Hence, at this point, our results represent a useful “safety-margin” analysis for non-invasive optical manipulations of *S. cerevisiae*. In our setup, the minimal power *P_c_* needed for stable 3-D confinement of the yeast cells was approximately 10 mW, well within the trapping power window where no significant increase in the cell mortality and generation time was observed. In this context, infrared optical tweezers can be viewed as a gentle, non-perturbing tool suitable for applications in cell biology. 

The design of our microfluidic chips with sets of identical cylindrical micro-chambers connected to the wide main channel by short, narrow side channels has proved very advantageous for our optical trapping experiments. When liquid is introduced into the chip for the first time, the chambers fill up slowly, but completely, due to the permeability of PDMS for gases and solubility of gases in the working liquid. Our system allowed for simple and fast cell isolation, optical manipulation, perfusion with various media, and unperturbed long-term observation for time periods up to 9 h, all while effectively preventing the cells from escaping. At the same time, the cells could be flushed from the chambers with a mild pressure pulse and the chip could be subsequently reused. We have used the same microfluidic chip for Raman spectroscopy of individual trapped yeast cells, observation of microalgae, diffusion experiments, etc. Thus, the presented versatile design might find multiple applications in various bio-microfluidic experiments.

## 4. Conclusions

In our study, we have used a specially designed microfluidic system to systematically investigate the increasing mortality and generation time and decreasing cell area index of *S. cerevisiae* cells subject to optical trapping with gradually increasing laser power. We have found a window of operating parameters (trapping power and time) within which the cells can be stably confined in an optical trap without detrimental effects on their physiological functions, as characterized by the unimpeded dynamics of cell division and accumulated growth of cellular mass. Our work aims to provide a benchmark for safe, non-invasive optical trapping of *S. cerevisiae*, which is one of the most important eukaryotic model organisms. Application of the presented results in the future optofluidic cell interrogation and sorting devices could improve the cell survival rate and help to achieve unbiased results, without artifacts due to the optical trapping. Microfluidic optical sorting of yeast cells assisted by fluorescence or Raman spectroscopy may become a crucial instrument for directed evolution of enzymes in yeast biotechnology; therefore, knowing the specific limits of optical micromanipulation is of utmost importance.

## Figures and Tables

**Table 1:** The summary of experimental results on the response of *S. cerevisiae* cells to light-induced stress (top section). Results reported by Aabo et al. in Ref. [20] (bottom section).

*P_c_* [mW]	*t_ot_* [min]	*E_c_* [J]	*µ_max_* [h^−1^]	DT [min]	DT [%]	GT [min]	GT [%]	M [%]	CAI(240)
0	15	0	0.3783	110	100	114	100	0	4.5
19	15	17	0.2940	141	129	115	101	0	3.7
38	15	34	0.2031	205	186	135	119	7	2.2
76	15	68	0.1487	280	254	259	228	50	1.7
95	15	86	0.0771	539	490	NA	NA	90	1.3
0 [20]	0	0	0.4284	97	100	92	100	NA	NA
1 [20]	50	3	0.3619	115	119	114	124	NA	NA
5 [20]	10	3	0.3500	119	123	121	132	NA	NA
10 [20]	5	3	0.3051	136	140	139	151	NA	NA

NA: Data not available.
